# Phenotypic spectrum in recessive STING-associated vasculopathy with onset in infancy: Four novel cases and analysis of previously reported cases

**DOI:** 10.3389/fimmu.2022.1029423

**Published:** 2022-10-06

**Authors:** Rensheng Wan, Johannes Fänder, Ia Zakaraia, Min Ae Lee-Kirsch, Christine Wolf, Nadja Lucas, Lisa Isabel Olfe, Corinna Hendrich, Danny Jonigk, Dirk Holzinger, Mathis Steindor, Gunnar Schmidt, Claudia Davenport, Christian Klemann, Nicolaus Schwerk, Matthias Griese, Brigitte Schlegelberger, Florian Stehling, Christine Happle, Bernd Auber, Doris Steinemann, Martin Wetzke, Sandra von Hardenberg

**Affiliations:** ^1^ Department of Human Genetics, Hannover Medical School, Hannover, Germany; ^2^ Institute of Anatomy and Cell Biology, Faculty of Medicine, Martin Luther University Halle-Wittenberg, Halle, Germany; ^3^ Department of Pediatrics, University Hospital and Medical Faculty Carl Gustav-Carus, Technische Universität Dresden, Dresden, Germany; ^4^ Institute of Pathology, Hannover Medical School, Hanover, Germany; ^5^ German Center for Lung Research, Biomedical Research in Endstage and Obstructive Lung Disease, Hannover, Germany; ^6^ Department of Pediatric Haemato-Oncology, University of Duisburg-Essen, Essen, Germany; ^7^ Department of Applied Health Sciences, University of Applied Sciences Bochum, Bochum, Germany; ^8^ Pediatric Pulmonology and Sleep Medicine, Cystic Fibrosis Center, Children’s Hospital, University of Duisburg-Essen, Essen, Germany; ^9^ Department of Pediatric Pneumology, Allergology and Neonatology, Hannover Medical School, Hannover, Germany; ^10^ Dr. von Hauner Children’s Hospital, Department of Pediatrics, German Center for Lung Research, University Hospital, Ludwig-Maximilians-University (LMU) Munich, Munich, Germany

**Keywords:** *STING1*, STING-associated vasculopathy of infancy, SAVI, pulmonary inflammation, interferonopathies, stimulator of interferon genes, STING

## Abstract

Gain-of-function variants in the stimulator of interferon response cGAMP interactor 1 (*STING1*) gene cause STING-Associated Vasculopathy with onset in Infancy (SAVI). Previously, only heterozygous and mostly *de novo STING1* variants have been reported to cause SAVI. Interestingly, one variant that only leads to SAVI when homozygous, namely c.841C>T p.(Arg281Trp), has recently been described. However, there are no entries in public databases regarding an autosomal recessive pattern of inheritance. Here, we report four additional unrelated SAVI patients carrying c.841C>T in homozygous state. All patients had interstitial lung disease and displayed typical interferon activation patterns. Only one child displayed cutaneous vasculitis, while three other patients presented with a relatively mild SAVI phenotype. Steroid and baricitinib treatment had a mitigating effect on the disease phenotype in two cases, but failed to halt disease progression. Heterozygous c.841C>T carriers in our analysis were healthy and showed normal interferon activation. Literature review identified eight additional cases with autosomal recessive SAVI caused by c.841C>T homozygosity. In summary, we present four novel and eight historic cases of autosomal recessive SAVI. We provide comprehensive clinical data and show treatment regimens and clinical responses. To date, SAVI has been listed as an exclusively autosomal dominant inherited trait in relevant databases. With this report, we aim to raise awareness for autosomal recessive inheritance in this rare, severe disease which may aid in early diagnosis and development of optimized treatment strategies.

## Introduction

The stimulator of interferon response cGAMP interactor gene, *STING1*, encodes a transmembrane protein STING (Stimulator of interferon response genes). STING is as a major regulator of innate immune responses against viral and bacterial pathogens (1). It is activated by cyclic GMP-AMP (cGAMP) which is produced by cyclic GMP-AMP synthase (cGAS) succeeding the recognition of aberrant double-stranded DNA (dsDNA). Following the binding of cGAMP, STING recruits TANK-binding kinase 1 (TBK1), which promotes TBK1 autophosphorylation. Subsequently, activated TBK1 phosphorylates the transcription factors interferon regulatory factor 3 (IRF3) which leads to induction of type I interferons (IFNs) and other IFN-stimulated genes (ISGs) (1–4). Type I IFNs then activate down-stream signaling pathways (e.g. the JAK-STAT pathway).

*STING1* gain-of-function (GoF) pathogenic variants (PVs) lead to the rare auto-inflammatory disease STING-associated vasculopathy with onset in infancy (SAVI; Online Mendelian Inheritance in Man no. 615934) (2). SAVI typically presents with systemic inflammation, recurrent fever, interstitial lung disease (ILD), cutaneous vasculopathy, and systemic inflammation with elevated C-reactive protein (CRP) and type I IFN activation. ILD typically occurs during childhood with tachypnea, hypoxia, and ground-glass opacities or fibrotic and bronchiectatic changes in chest computed tomography (CCT) imaging. Treatment is challenging and aims to suppress the activated inflammatory cascade and to inhibit uncontrolled type I IFN activation (e.g. with JAK1/2 inhibitors) (5–7). Since its first description in 2014 (2), more than 20 cases with autosomal dominant SAVI have been described. A total of 16 different heterozygous, causative missense variants in *STING1* have been reported, most of which have arosen *de novo* (2, 8, 9). Unfortunately, most of them are not listed in public databases (e.g., ClinVar) (10). In addition, complex alleles with two PV *in cis*, causing an additive gain-of-function, have been described (11, 12). Recently, a homozygous missense *STING1* PV following autosomal recessive inheritance have been reported (13, 14). In public databases such as OMIM, however, SAVI remains to be listed as an exclusively autosomal dominant disease (15). This may pose a challenge for treating physicians and genetic laboratories in the diagnostic process.

Here, we report on three patients of three unrelated families harboring PV c.841C>T p.(Arg281Trp) in *STING1* (NM_198282.3) that only causes SAVI when homozygous. While four patients showed SAVI features including ILD, tachypnea and hypoxia, failure to thrive, and increased IFN activation, only one of them displayed skin vasculitis - a hallmark phenotypic feature of SAVI. We present comprehensive data on all thus far published homozygous c.841C>T p.(Arg281Trp) SAVI cases and demonstrate that it is essential to consider an autosomal recessive (AR) inheritance pattern in *STING1* in a patient with inflammatory interstitial lung disease, even in the absence of acral vasculopathy.

## Methods

### Patients

Newly described SAVI cases were recruited through Hannover Medical School´s Department of Pediatric Pneumology, Allergology and Neonatology and through Department for Pediatric Pulmonology and Sleep Medicine, University of Duisburg-Essen and were enlisted in the European childhood interstitial lung disease registry network (ChILD-EU). Before enrollment, informed written consent was obtained from the subjects and/or their legal guardians. Genotyping was performed at Hannover Medical School´s Department of Human Genetics.

### Sample preparation

DNA was extracted from EDTA-whole blood samples using the NucleoMag Blood kit (Macherey-Nagel, Düren, Germany). DNA quality was measured with the Qubit^®^ Fluorometer using the Qubit dsDNA BR Assay Kit (Life Technologies, Darmstadt, Germany).

### Whole exome sequencing

Whole exome sequencing (WES) was conducted with the IDT Exome library kit (xGen, IDT, Leuven, Belgium). An Illumina NextSeq 500 sequencer (Illumina, San Diego, California, USA) was used to sequence captured DNA to generate 150-bp paired-end reads. Sequencing was conducted according to the manufacturer’s instructions. Reads were aligned to the human reference genome GRCh37/hg19. A mean coverage of 150 reads (on target read ~98.5%) with the targeted region covered by at least 20 reads was achieved.

### Whole genome sequencing

Whole genome sequencing (WGS) was performed using the polymerase chain reaction (PCR)-free Lotus DNA Library Prep Kit (IDT, Leuven, Belgium) with an insert size of ~350 bp. DNA was sequenced using an Illumina NovaSeq 6000 Sequencer (Illumina, San Diego, California, USA) to generate 160-bp paired-end reads. Sequencing was performed following the manufacturer’s instructions. Reads were aligned to the human reference genome GRCh37/hg19. A mean genome-wide coverage of 40x (on-target read ~94%) was achieved, with the target region covered by at least 20 reads.

### Sequence variant analysis

The Medical Genetics Sequence Analysis Pipeline (megSAP) was used for next-generation sequencing (NGS) data analysis (16). Reads from WES and WGS were aligned to the human reference genome GRCh37/hg19. Single nucleotide variants (SNVs) were filtered for location in exonic and splice site regions of genes (+/- 20 bases). Deeper intronic variants were excluded from the analysis. We only analyzed rare variants with a minor allele frequency (MAF) of <=1% in the 1000 Genomes project database or the genome aggregation database (gnomAD) (17). SNVs were annotated with Alamut Visual, version 2.12 (Interactive Biosoftware, Rouen, France) and visualized with the Integrative Genomics Viewer (IGV), version 2.5.3 (18). In order to get information about the variants’ pathogenicity, we used prediction tools, including phyloP (deleterious threshold >1.6) (19), SIFT (20), PolyPhen-2 (21), FATHMM (22), CADD (deleterious threshold >=20) (23) and REVEL (24).In addition, LOVD (https://databases.lovd.nl/shared/), ClinVar (https://www.ncbi.nlm.nih.gov/clinvar), and gnomAD were screened for entries of identified variants. IGV was used to manually review variants with insufficient coverage or poor quality. The interpretation of sequence variants was based on the standards and guidelines of the American College of Medical Genetics and Genomics (ACMG) (25).

### Sanger sequencing

The segregation analysis for family 1 was conducted by Sanger sequencing. Primers were designed with the genomic sequence *STING1* (NG_034249) for exon 7 and the flanking two introns. The forward primer was 5′-GGACCCTCCATTCTCCATC-3′, and the reverse primer was 5′-GGTCTCCCAAAGAGTCAGAAG-3′. The variant was amplified with the genomic DNA by PCR. The amplified products were purified (MinElute 96 UF PCR Purification Kit, QIAGEN, Hilden, Germany) and cycle-sequenced using fluorescent dye-termination (BigDye Terminator v1.1 Cycle Sequencing Kit, Applied Biosystems, Darmstadt, Germany) and an ABI 3100 or ABI 310 automatic capillary genetic analyzer (Applied Biosystems, Darmstadt, Germany) in both forward and reverse directions.

### Real-time quantitative (rtq) PCR of IFN-stimulated genes

IFN scores to describe type I IFN activation in our patients were analyzed as described elsewhere (26). In brief, total RNA was isolated from peripheral blood mononuclear cells (PBMCs) separated by Ficoll density gradient centrifugation with the RNeasy Mini Kit (Qiagen, Hilden, Germany) followed by DNase I digestion. Real-Time qRT-PCR was conducted with the Taqman Universal PCR Master Mix on an ABI 7300 PCR machine (Applied Biosystems, Darmstadt, Germany). IFN scores were calculated from the median fold change in relative mRNA expression of seven IFN-stimulated genes (*IFI27*, *IFI44*, *IFI44L*, *IFIT1*, *ISG15*, *RSAD2*, *SIGLEC1*) normalized to *HPRT* and *GAPDH* as previously described (27). The following threshold were applied: <12.49: no increase in IFN signature; 12.49-30, weak IFN activation; 30-60, moderate IFN activation; 60-200, strong IFN activation; >200, very strong IFN activation (27).

## Results

**Figure 1:**
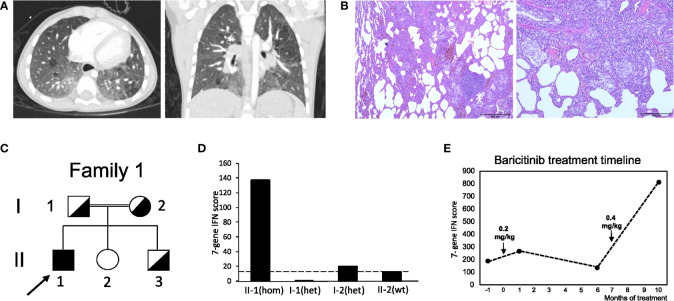
Clinical features and pedigree of patient 1. **(A)** Chest CT at the age of five years, displaying milk glass opacities, cystic lesions and architectural distortion consistent with interstitial fibrosis. **(B)** Lung biopsy displaying interstitial hemorrhage. **(C)** Pedigree of patient 1’s family; filled symbol: homozygosity; half-filled symbol, heterozygosity; clear symbol: wild type; arrow indicates patient 1 (II-1). **(D)** IFN score from whole blood for the patient with c.841C>T p.(Arg281Trp) homozygosity (hom) after receiving baricitinib treatment at month 6 and his healthy family members carrying the variant in heterozygosity (het) or wild type (wt) *STING1* alleles; dash line indicates threshold of 12.49 from healthy controls. **(E)** IFN score changes in patient 1 before and after baricitinib treatment. Patient started to receive baricitinib treatment at month 0 and dosage was increased at month 7.

The first case (patient 1) was a boy born after normal pregnancy to consanguineous parents of Syrian origin. He had one healthy sister, and no further cases of lung disease were reported by the family. Starting from the age of five months recurrent lower airway infections with fever and hypoxia occurred. At the age of nine months the patient first suffered from hypoxic seizures which occurred one to five times a day after crying with cyanosis of lips and hands, followed by loss of consciousness for around thirty seconds, closure of eyes and short, clonic limb movements. Psychomotor development was normal, and although the boy was rather small and slender, body height and weight developed normally. Since the patient´s first year of life, the parents had also noted tachypnea during sleep. At the age of three years, the family migrated to Germany. Because the seizures persisted to occur daily, psychomotor and electro encephalogram (EEG) analyses were conducted shortly after the patient´s third birthday. Results were normal. Treatment with sulthiame was initiated, but had no effect. At this age, the boy presented with chronic tachypnea with about 60 breaths per minute (brpm), dry cough, jugular retractions, reduced exercise tolerance, and clubbing of fingers. Cutaneous pathologies were absent. A chest radiograph revealed bilateral infiltrates. One month later, chest computed tomography (CCT) was performed, which showed bilateral infiltrates and milk glass opacities in both lower and upper lobes. Sweat testing and immunological analyses ruled out cystic fibrosis and tuberculosis. Transcutaneous oxygen saturation (SpO2) was measured during the seizures, which then dropped to approximately 50%. Sulthiame was discontinued based on the diagnosis of hypoxic seizures. The boy was referred to our center at the age of 3.5 years. At this time, the boy was in the low but normal range for body weight (13 kg, -1.5z) and height (93 cm, -1.9z). While echocardiography, EEG, and abdominal ultrasound were normal, laboratory analyses revealed mild leukocyte elevation (16.3 G/nl) and increase in serum IgG (22.9 g/l), IgE (434 IU/ml), and antinuclear antibodies (ANA, 1:5120). cANCA and pANCA antibodies were positive. CCT revealed bilateral milk glass opacities and subpleural cystic lessions. Bronchoscopy showed normal airway anatomy, but mild granulocytic inflammation and increased siderosis of bronchoalveolar lavage fluid (BALF) macrophages indicated alveolar hemorrhage. No bacterial or viral pathogens were detected in the BALF samples. Lung biopsies from the left upper and lower lobe were obtained, which revealed severe and diffuse interstitial hemorrhage (**Figure 1B**). Based on the disease classification as “diffuse interstitial lung disease with immune dysregulation”, treatment with hydroxychloroquine (10 mg/kg p.o. daily) and monthly systemic pulses of high-dose methylprednisolone (three days 15mg/kg methylprednisolone i.v.) were started. After three months of this treatment, marked improvement with significant reduction of tachypnea (30-40 brpm), reduced dry cough, improved exercise tolerance, and almost complete absence of hypoxic seizures was observed. Methylprednisone pulses were stopped, and the boy remained on daily oral hydroxychloroquine treatment. The initial genetic analysis revealed no suspicious finding (*ABCA3, CSF2RB, FLNA, FOXF1, NKX2-1, SFTPB, SFTPC, TBX4, COPA*). Upon re-evaluation at the age of four years, one year after initiation of hydroxychloroquine therapy, body height and weight were within the normal range (15 kg, -1.3z, height 102cm (-1)). Comparably mild tachypnea (44 brpm), and dry cough, dyspnea and jugular retractions occurred only during physical exercise. SpO2 levels measured during exercise and during sleep as well as echocardiography were normal. Serum IgG and ANA levels remained significantly elevated, and a marked increase in IFN activation was noted. Reevaluation of sequencing results now detected a pathogenic variant (PV) in *STING1* c.841C>T p.(Arg281Trp), and both healthy parents and one sibling were heterozygous for this mutation (**Figure 1C** **;** **Figure S1A**). Due to the diagnosis of SAVI-associated ILD, a new round of monthly high-dose methylprednisone pulses over a period of three months was administered, and treatment with the JAK inhibitor baricitinib (0.2 mg/kg) was added shortly after the boys fifth birthday. At this time, IFN scores were significantly elevated in the patient, but not in any other family members (**Figure 1D** **;** **Table S1**). Screening for autoantibodies was negative. Six months after initiation of this treatment, the boy´s condition stabilized. His breathing rate at rest was 26 brpm, and he was able to walk distances up to 2,000m before dry cough or dyspnea started. He was able to walk 430 m at this time (significantly reduced as compared to healthy age-matched children) with no pathological drop in SpO2 levels. Echocardiography, EEG, ophthalmological examination, audiometry, psychomotor and body weight and height development, and skin at this time point were normal. However, CCT at this time point showed increase of signs of fibrosis with cystic lesions, reduced volumes of both lower lobes, architectural distortion as well as diffuse ground glass opacities (**Figure 1A**). BALF analyses showed mild granulocytic inflammation and increased siderosis of alveolar macrophages. In addition, the IFN signature was still significantly elevated (**Figure 1E** **;** **Table S1**), and baricitinib doses were increased (0.4 mg/kg). At the last follow up shortly after the boys sixth birthday, the boy showed normal body height and weight (20 kg, -0.9z, 114 cm (-0.2z)) and normal psychomotor development, but again significantly reduced walking distance in the six-minute walk test (423 m) with borderline drop in SpO2 levels during exercise (min 92%). First pulmonary function testing indicated restrictive lung pathology. Echocardiography again was normal. Baricitinib doses were slightly increased (0.5 mg/kg).

**Figure 2:**
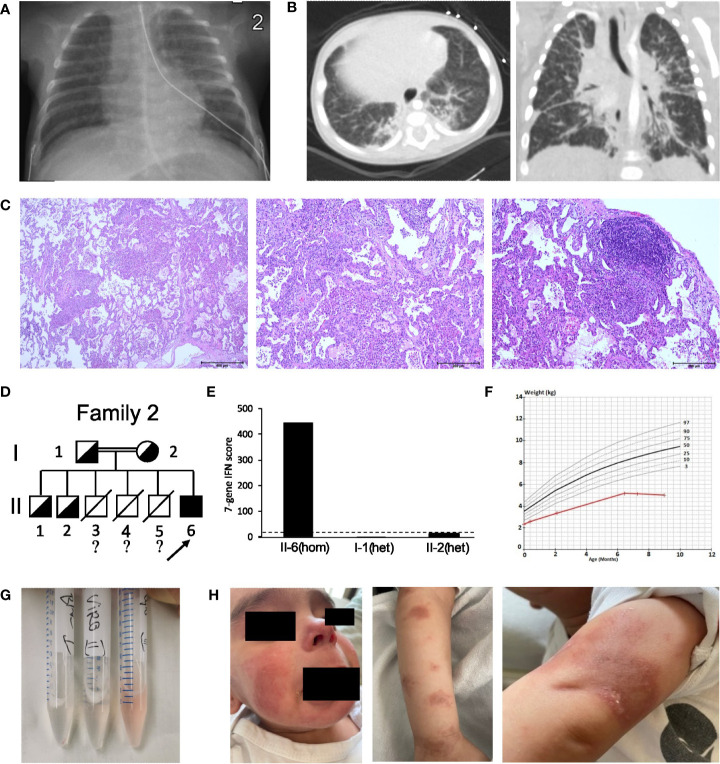
Clinical features and pedigree of patient 2. **(A)** Chest X-ray of the newborn patient, displaying therapy-refractory perihilar streaky consolidations. **(B)** Low dose chest CT at the age of five month revealed fibrotic changes with traction-bronchiectasis, thickened interlobular and intralobular septa, and architectural distortion. **(C)** Lung biopsy displaying parenchymal hemorrhage, interstitial glycogenosis, lymphofollicular hyperplasia and accumulation of alveolar macrophages. **(D)** Pedigree of patient 2’s family; filled symbol: homozygosity; half-filled symbol, heterozygosity; clear symbol: wild type;?, no information about genotype; slash, died individuals; arrow indicates patient 2 (II-6). **(E)** IFN score from whole blood for patient 2 with c.841C>T p.(Arg281Trp) homozygosity (hom) and his healthy family members carrying c.841C>T p.(Arg281Trp) in heterozygosity (het); dash line indicates threshold of 12.49 from healthy controls. **(F)** Weight vs. Age chart for patient 2, displaying failure to thrive. Red line, patient 2; black and grey lines, standard weight-for-age percentiles for 0-12 months breast-fed boys. **(G)** Sequential bronchoalveolar lavage (BALF) samples indicating alveolar hemorrhage. **(H)** SAVI characteristic, cutaneous vasculitis of the face and arms.

The second case (patient 2) was a boy born prematurely at 36 + 4 pregnancy weeks in Germany to consanguineous parents of Syrian origin. He had two healthy brothers and three deceased brothers who had died in infancy from recurrent infections. Due to persisting dyspnea, tachycardia and systolic murmur, the patient was referred to our center at the age of 12 days, when he presented as floppy and dystrophic newborn (body weight 2.3 kg), and displayed persisting tachypnea (60-70 brpm) with hypocapnia and tachycardia (180 bpm). Telangiectasia on the cheeks and fine-spotted erythema of the chest were noted. Laboratory testing revealed elevated interleukin (IL)-6 (40 pg/ml) and NT-pro-BNP (>3000 ng/l), hypocalcemia, increased serum inorganic phosphate and mild thrombocytosis. All other blood tests including further electrolytes, kidney, liver and vitamin D status, parathyroid hormone, and other blood counts were normal. Chest X-ray (CXR) revealed fine-spotted pulmonary infiltrates and streaky consolidations (**Figure 2A**). Echocardiography showed a small ventricular septal defect with normal cardiac function and no signs of pulmonary hypertension. Audiometry revealed slight hearing loss on the right side. Further diagnostic workup including newborn screening and extended metabolic investigations, ECG, EEG, sonography of abdomen, brain, thymus, and hips as well as ophthalmologic examinations were normal. Microbiological analyses ruled out common airway pathogens including tuberculosis and pertussis. Treatment with piperacillin/tazobactam and clarithromycin was started, but tachypnea and streaky consolidations in the CXR persisted, leading to chest computed tomography (CCT) at the age of one month. Here, congenital interstitial lung disease displayed signs of fibrotic scarring with traction-bronchiectasis, architectural distortion and reticulation (**Figure 2B**). As telangiectasia and skin rash, as well as tachypnea and failure to thrive persisted, nutrition with amino acid-based formula milk and treatment with inhaled corticosteroids were started. This led to slight improvement in weight gain but had no other significant effect. At the age of four months, the boy received intermittent oxygen supplementation up to 0.5 L/min, and follow up CCT confirmed fibrotic lung consolidations with bronchiectasis, milk glass opacities, and thickening of interlobar septi. Bronchoscopy was normal at this time. Thoracoscopic lung biopsies were taken, and histological analyses revealed signs of parenchymal hemorrhage, interstitial glycogenosis, lymphofollicular hyperplasia and accumulation of alveolar macrophages consistent with diffuse interstitial pneumonitis. However, fibrosis could not be confirmed histologically (**Figure 2C**). WGS detected the homozygous PV c.841C>T p.(Arg281Trp) in *STING1*, which was also present in heterozygosity in the patient´s parents and two elder siblings (**Figure 2D** **;** **Figure S1B**). The IFN score was significantly increased in the patient, but not in the other healthy family members (**Figure 2E** **;** **Table S1**). Systemic steroid treatment (three days 15 mg/kg prednisolone i.v.) was started, which lead to an intermittent but not sustained reduction of additional oxygen supplementation and significant improvement of the skin rash. At the age of seven months, anti-DNA (12 IU/ml) and anti ENA ss-A/ro antibodies (20 IU/ml) were positive, and the boy remained severely dystopic (body weight 5.01 kg, -3.9z, height 65 cm (-2.6z) (**Figure 2F**)) and presented with impaired psychomotor development. BALF analyses indicated alveolar hemorrhage (**Figure 2G**). ILD-related features had progressed, and teleangiectasia of cheeks also persisted, with progressive cutaneous necrotizing vasculitis on the boy’s chest, hands, and feet with necrotic lesions (**Figure 2H**). Echocardiography revealed normal biventricular function without signs of pulmonary hypertension, while CXR showed bilateral infiltrates. Due to persistent tachypnea and skin rash, another steroid course (three days 15 mg/kg prednisolone i.v.) was given at the age of nine months, which again showed beneficial effects on the skin rash but had no effect on ILD-related symptoms. Treatment with the JAK inhibitor ruxolitinib (1 mg/kg p.o. daily) was started. At the last follow-up shortly before the boys first birthday, he remained severely dystrophic [body weight 5.6 kg, -4.2z, height 70 cm (-2.3z)] with impaired psychomotor development. Oxygen supplementation was needed. Magnetic resonance imaging of the brain revealed T2 enhancement of myelin structures and borderline wideness of ventricles. The boy remains to be treated with ruxolitinib (currently 1 mg/kg p.o. daily).

**Figure 3:**
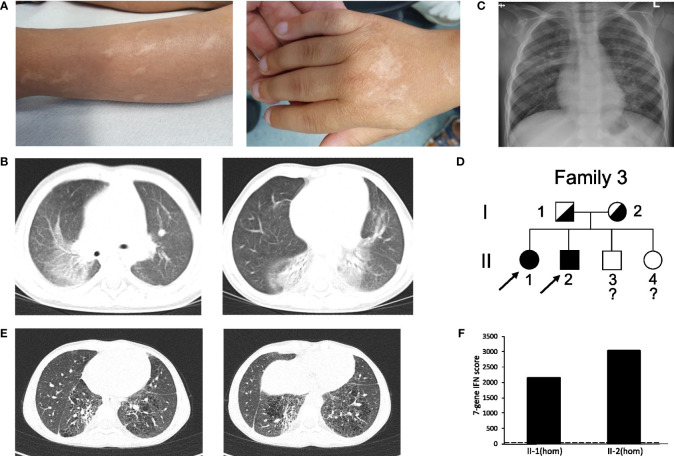
Clinical features and pedigree of patient 3 and 4. **(A)** scars of the SAVI skin disease during infancy without activity in patient 3. **(B)** CT of patient 3 at the age of three years displaying ground glass opacities, atelectasis, interlobar septal thickening and cystic changes. **(C)** Chest X-ray of the patient 3, displaying bilateral interstitial lung disease and depended dystelectasis. **(D)** Pedigree of patient 3 and 4’s family; filled symbol: homozygosity; half-filled symbol, heterozygosity; clear symbol: unaffected;?, no information about genotype; arrow indicates index patient 3 (II-2) and 4 (II-1). **(E)** CT of patient 4 showing fibrotic and cystic lesions. **(F)** IFN score from whole blood for patient II-2 and II-1; homozygosity (hom); dash line indicates threshold of 12.49 from healthy controls.

The third case (patient 3) is a boy born after normal pregnancy to non-consanguineous parents, who originate from the same village in Syria. He had two sisters and one brother. The family migrated from Syria *via* Greece and was accommodated in several German cities before he presented to our department. In Syria at the age of one year the boy suffered from a significant presumed bullous skin disease affecting the extremities and the face but sparing the trunk. After seven months the skin disease healed completely without specific therapy, but with residual scars (**Figure 3A**) without further activity in the recent years. Respiratory problems resembling respiratory tract infection (including one documented RSV infection) were reported at the age of three years. Furthermore, an arthritis was reported when located in Greece which led to the suspicion of an immunologic disorder (“congenital systemic lupus erythematodes”) and an immunosuppressive combination therapy with mycophenolate mofetil, steroids (unknown dose) and azithromycin was initiated. With this therapy the patient improved and returned to normal activity. During the further course RSV bronchitis was diagnosed, which led to the discontinuation of the immunosuppressive therapy. Because of clubbing, additional diagnostics were initiated. Chest computed tomography (CCT) showed bilateral ground glass opacities (**Figure 3B**), normal values were recorded for oxygen saturation, complete blood count, liver and kidney parameters, serum amyloid A, subclasses of lymphocytes, and negative result for tuberculosis test. Pathologic values were reported for serum IgG (17.8 g/l), IgE (630 IU/ml), antinuclear antibodies (ANA, 1:3200), p-ANCA (MPO-ANCA 7.8 U/ml) and rheumatoid factor (20 IU/ml). Discontinuation of immunosuppressive therapy resulted in adynamic state and pain of the lower extremities lacking signs of arthritis.

In our department we saw an adynamic child (14.3 kg, z-score -2.20, height 97 cm; z-score -2.76), with clubbing but no overt hypoxemia. He was only able to walk short distances. Blood count showed a thrombocytosis and hypochrome, microcytic erythrocytes. Parameters for systemic inflammation were only mildly elevated (CRP 0.7 mg/dl, C3 1.6 g/l, C4 0.53 g/l, sIL2-R 912 U/ml). Furthermore, pathologic laboratory values were reported for serum IgG (24.8 g/l), IgE (385 IU/ml), IgM (2.29 g/l), IgA (5.5 g/l) and antinuclear antibodies (ANA > 1:5120 without specific ENA), ANCA (1:1280), (MPO-ANCA 128 U/ml) and rheumatoid factor (20 IU/ml). Echocardiography was normal, chest X-ray showed bilateral interstitial lung disease (**Figure 3C**). After initiation of a methylprednisolone pulse (20 mg/kg for three days) the adynamic state and lower extremity pain normalized, but returned after only two weeks. Subsequent genetic testing detected the homozygous PV in *STING1* c.841C>T p.(Arg281Trp) (**Figure 3D** **;** **Figure S1C**). One of his sisters (patient 4) with ILD, clubbing and failure to thrive (19.9 kg, z-score -2.35, height 115 cm; z-score -2.87) was also diagnosed with the same homozygous PV in *STING1* (**Figure 3D** **;** **Figure S1C**). Chest CT showed fibrotic and cystic lesions (**Figure 3E**). Parameters of chronic inflammation were mildly elevated (SIL-2R 1115 U/ml, IgG 223.7 g/l, IgM 2.14 g/l, ANA >1:5120 without specific ENA). IFN score was highly elevated in those two patients (**Figure 3F**).

To corroborate our finding on recessive inheritance in SAVI, we screened the literature on further cases, unrelated to our patients. In addition to our report, eight additional patients with SAVI due to homozygous *STING1* c.841C>T p.(Arg281Trp) from five further families have been described recently (13, 14) (Patient 5-12 in **Table 1**). Information on the age-of-onset was available in six of the eight cases and the mean age at first symptoms was four months (Stdev 1.6; range 1-8). Lin et al. have described six children who had symptoms of SAVI with onset in infancy (Patient 5-10). The dominant clinical features included ILD (in five patients, 83%), failure to thrive (in five patients, 83%), clubbing (in four patients, 67%), and cough (in four patients, 67%). Symptoms of recurrent fever, recurrent infections, vomiting, diarrhea, short stature, pulmonary hypertension, polyarthritis, and hair loss have also been described in this report. Five of the patients showed skin manifestations such as facial or plantar, cutaneous necrotizing vasculitis (**Table 1**). With regard to treatment, barticitinib was predominantly chosen (three patients) while one patient was treated with ruxolitinib. Treatment responses are noted in **Table 2**. One patient with early onset SAVI received the JAK inhibitor tofacitinib, but passed away at the age of five months. Another patient with cough and failure to thrive passed away at 18 months of age without any prior anti-inflammatory treatment (**Table 2**). IFN scores were elevated in classic AD SAVI, as well as in patients carrying homozygous *STING1* c.841C>T p.(Arg281Trp), but not in healthy family members who were heterozygous carriers of the variant (13). Alghamdi et al. have reported two siblings from another consanguineous family carrying *STING1* c.841C>T p.(Arg281Trp), in which two family members homozygous for this variant displayed pulmonary hypertension and ILD (**Table 1**, Patient 11 and 12). Both of them also presented with facial vasculitic skin rash. Also in this family, dyspnea, recurrent airway infections, clubbing, and hair loss were reported. Elevation of gene expression and plasma levels of IFN-β1 was observed in homozygous carriers compared to healthy heterozygous or wild type family members in this family, again indicating the constitutive activation of the STING pathway only in homozygous carriers of this variant. After receiving ruxolitinib, symptoms have been reported to improve in both cases (**Table 2**) (14).

**Table 1:** Demographical and clinical features of AR SAVI in newly identified cases (patient 1-4) and cases from the literature [cases 5-12; (13, 14)].

Patient No.	Gender	Ethnicity	Age at onset of symptom	Age at last evaluation	Clinical features	Cutaneous features	Auto-antibodies	Laboratory markers
**1**	Male	Arabic	5 months	6 years	ILD, diffuse alveolar hemorrhage, recurrent severe hypoxaemia, tachypnea, clubbing	none	ANA + ANCA +	ESR, IgG, IgE ↑
**2**	Male	Arabic	12 days	11 months	ILD, lung fibrosis, bronchiectasis, hypocalcemia, delayed psychomotor development, acidosis, failure to thrive	facial telangiectatic lesions/rash	anti DNA+ anti ENA+ (anti ENA ss-A/ro)	CRP, IgA, IgG1, IgG2↑
**3**	Male	Arabic	12 months	3 years	ILD, clubbing, skin disease, failure to thrive, adynamie, arthritis	suspected vasculitis lesion during infancy	ANA + ANCA +	IgG, IgM, IgA, IgE ↑
**4**	Female	Arabic	unknown	8 years	ILD, clubbing, failure to thrive	none	ANA +	IgG, IgM ↑
**5**	Male	Arabic	4 weeks	5 months	ILD, diffuse bilateral parenchymal opacities, cough, failure to thrive	a livedoid appearance rash	ANA +	CRP ↑
**6**	Male	Arabic	unknown	unknown	Chronic cough, failure to thrive	unknown	unknown	unknown
**7**	Male	Arabic	3 months	unknown	ILD, alveolar hemorrhage, cough, failure to thrive, vomiting, diarrhea, clubbing, recurrent fever	erythematous rash	ANA + ANCA + (anti PR3)	CRP, ESR, IgG, IgA, IgM↑
**8**	Male	Arabic	6 months	unknown	ILD, delayed psychomotor development, failure to thrive, clubbing	facial rash	ANA + ANCA + (anti MPO) anti DNA +	CRP, ESR, IgG ↑
**9**	Female	Arabic	2 months	unknown	suspicious for ILD, pulmonary hypertension, recurrent lung infections, tachypnea, polyarthritis, clubbing	plantar erythematous rash	ANA + ANCA +	CRP, ESR, IgG ↑
**10**	Male	Arabic	8 months	unknown	suspicious for ILD, cystic lung lesions, fibrosis, failure to thrive, polyarthritis, hair loss, clubbing	intermittent vasculitic rashes	ANA + ANCA +	CRP, ESR, IgG, IgM ↑
**11**	Male	Arabic	unknown	15 years	suspicious for ILD, pulmonary hypertension, Raynaud’s phenomenon, emphysema, recurrent chest infections, clubbing	violet-red skin rash	ANA +	CRP, ESR, IgA, IgG ↑
**12**	Female	Arabic	Toddler years	7 years	suspicious for ILD, diffuse bilateral opacities, pulmonary hypertension, arthritis, Raynaud’s phenomenon, hair loss	malar, facial rash	ANA + ANCA + anti DNA +	ESR, IgA, IgG ↑

ILD, interstitial lung disease; ANA, antinuclear antibody; ANCA, antineutrophil cytoplasmic antibody; anti ENA ss-A/ro: anti extractable nuclear antibodies; anti-PR3, anti-proteinase 3 antibody; anti-MPO, anti-myeloperoxidase; c-ANCA, cytoplasmic antineutrophil cytoplasmic antibody; dsDNA, double-stranded DNA; CRP, C-reactive protein; ESR, erythrocyte sedimentation rate; Ig, Immunoglobulin; +, positive.

**Table 2:** Treatment regimens and therapy responses in AR SAVI in newly identified cases (patient 1-4) and cases from the literature [cases 5-12; (13, 14)].

PatientNo.	Treatment(dosage)	Duration of JAK-I treatment	IFN score before treatment	IFN score during treatment	dermatological pathologies	Oxygen dependance	CT	Summary
**1**	initially hydroxychloroquine (10mg/d/kg) orally, steroid pulse therapy, then baricitinib, up to 0.5 mg/kg p.o. daily	15 months	371; 564; 186 (7-gene IFN score)	264; 137; 809 (7-gene IFN score)	none	intermittent O2 supplementation, mitigating effect of HCQ, steroids, and baricitinib on pulmonary phenotype suspected	progression of pulmonary fibrosis	baricitinib and steroids may have mitigating effect on disease progression, but progression occurred in spite of treatment
**2**	steroid-pulse therapy, then ruxolitinib up to 1 mg/kg p.o. daily	2 months	317; 444 (7-gene IFN score)	N/A	positive effect of steroid pulse on skin lesions	intermittent O2 supplementation, no effect of steroids on pulmonary phenotype; Mitigating effect of ruxolitinib on pulmonary phenotype suspected	unknown	steroids ameliorated skin but not lung features of SAVI. Ruxolitinib may have mitigating effect on disease progression, but progression occurred in spite of treatment.
**3 **	steroid pulse therapy	N/A	3024 (7-gene IFN score)	N/A	N/A	no	N/A	steroids improved adynamic state and arthralgia
**4**	none	N/A	2135 (7-gene IFN score)	N/A	N/A	no	N/A	therapy not started yet
**5**	steroid therapy, short-term treatment with tofacitinib	N/A	N/A	N/A	N/A	N/A	N/A	†patient died due to respiratory failure at 5 months of age
**6**	none	N/A	600 (28-gene IFN score)	N/A	N/A	N/A	N/A	†patient died due to failure to thrive at 18 months of age
**7**	ruxolitinib	N/A	100 (28-gene IFN score)	N/A	N/A	N/A	N/A	beneficial
**8**	steroid treatment, baricitinib	N/A	N/A	N/A	N/A	N/A	N/A	beneficial
**9**	baricitinib at the age of 7 years	N/A	N/A	N/A	N/A	oxygen dependence continued	N/A	beneficial and clinical improvement
**10**	baricitinib	N/A	N/A	N/A	N/A	N/A	N/A	beneficial
**11**	systemic steroid treatment, then ruxolitinib	6 months	81.8 pg/ml (IFN β1 plasma level)	10.9 pg/ml (IFN β1 plasma level)	positive effect of ruxolitinib on skin lesions	positive effect of ruxolitinib on pulmonary phenotype	no detailed information provided	beneficial and clinical improvement of ruxolitinib
**12**	steroid treatment, methotrexate, hydroxychloroquine, then ruxolitinib	N/A	70 pg/ml (IFN β1 plasma level)	N/A	N/A	positive effect of ruxolitinib on pulmonary phenotype	N/A	clinical improvement of ruxolitinib: reduction in the size of the area of hair loss with hair regrowth and a decrease in the hypopigmentation.

mg/kg/d, milligram per kilogram per day; IFN, interferon; N/A, not applicable.

## Discussion

In this study, we present four newly diagnosed, unrelated patients carrying the homozygous, pathogenic GoF-variant c.841C>T p.(Arg281Trp) in exon 7 of *STING1* causing AR SAVI. All patients suffered from severe ILD. While one child also presented with failure to thrive, delayed psychomotor development, and typical cutaneous vasculitis, the other three patients displayed a comparably mild phenotype without skin rash. In summary, however, four patients displayed hallmark findings of SAVI (2), and all children were severely ill. Sequencing did not identify additional variants that could explain the phenotype. c.841C>T p.(Arg281Trp) has previously been reported in heterozygosity in two of 282,822 alleles in the gnomAD population database. It was predicted as “deleterious” by SIFT, “probably damaging” by PolyPhen2, “disease causing” by MutationTaster, and the CADD score was 26.2. The REVEL score was 0.27. In summary, we classified this variant as “pathogenic” (class 5) according to ACMG guidelines using the criteria PS3, PM1, PM2 and PP3 (25). In addition to these novel cases, we performed a thorough literature review corroborating our finding on AR inheritance in SAVI. We present eight further cases that suffer from AR SAVI due to homozygosity of c.841C>T p.(Arg281Trp).

Our findings are important in several aspects. First, we clearly show that SAVI can follow an AR inheritance pattern and, moreover, by a GoF variant. It is rather rare that pathogenic GoF variants follow an AR inheritance pattern and only a few genes have been described so far, for example *NLRP1* and *STAT2* (28, 29). The observation that a monogenic disease may be due to variants affecting either only one or both alleles of one gene is uncommon. Typically, this occurs in diseases following AD inheritance due to compound heterozygosity (30, 31). Also for SAVI, complex allelic traits with two PV *in cis*, leading to additive gain of function, were described (11, 12). For example, for adult-onset polycystic kidney disease, which is typically an AD trait, homozygosity of hypomorphic variants in the polycystic kidney disease 1 gene *PKD1* may be disease causing, while heterozygous mutations lead to a mild phenotype with only minor renal cysts, altogether rather advocating for incomplete penetrance than for true AR inheritance (32). Importantly, neither in the families newly identified by us nor in the literature, heterozygous carriers of c.841C>T p.(Arg281Trp) displayed any SAVI-associated clinical pathology, and there were no signs of IFN activation in any heterozygous family members in all of the seven families (13, 14), supporting the notion that c.841C>T p.(Arg281Trp) truly causes AR SAVI.

It is important to note that c.841C>T p.(Arg281Trp) in *STING1* was classified as “of uncertain significance” by Invitae in March 2019 and GeneDx in April 2020 (Accession: SCV001205296.3 and Accession: SCV001823939.1). As of today, we have classified this variant as “pathogenic” in ClinVar, but it is still not updated by Invitae and GeneDx and there is still no entry in LOVD. An AR inheritance pattern of SAVI is still not registered in OMIM, which might complicate the correct diagnosis for genetic laboratories and delay the treatment. A timely notification for those public databases is important to facilitate patients’ management, and is recommended. Considering the high mortality and poor prognosis of SAVI, we strongly recommend testing for *STING1* variants and considering an AR inheritance pattern in all children with a suggestive clinical phenotype.

The mechanism behind enhanced inflammation in c.841C>T p.(Arg281Trp) associated AR SAVI is not entirely clear. The physicochemical difference between these amino acids is that glutamine is polar and hydrophilic, whereas tryptophan is nonpolar and hydrophobic (33), which may lead to distinct changes in protein conformation and binding. The physiological arginine at position 281 in *STING1* is highly conserved and present in most species (34), and the chemical difference between arginine tryptophan [as induced by c.841C>T p.(Arg281Trp)] is moderate (Grantham distance 101). Lin et al. have shown that transfection of HEK293T cells with the *STING1* construct including the homozygous variant c.841C>T p.(Arg281Trp) has led to an increase in IFNB1 reporter activity in the absence of ligand binding. Moreover, the mutant remained responsive to the induction of cGAMP, which indicates that the amino acid change has no effect on ligand binding (13). Normally, inhibitors bound to the polymer interface of the wild-type STING, prevent polymerization and autoactivation. However, homozygosity of c.841C>T p.(Arg281Trp) appears to lead to an auto-activation of STING due to the inability of inhibitors to bind at the STING interface where p.(Arg281) locates (13). Another hypothesis is that c.841C>T p.(Arg281Trp) induces a conformational change in STING, enabling ER-Golgi trafficking and TBK1/IRF3 phosphorylation without cGAMP binding. *In vitro* experiments by Lin et al. have shown, however, that c.841C>T p.(Arg281Trp) results in reduced auto-activation than classical variants leading to autosomal dominant SAVI, supporting the notion that this variant is a hypomorphic gain-of-function variant (13). Thus far no other *STING1* variants are known to cause AR SAVI.

Interestingly, a variant inducing a different amino acid change at the same position as in our newly identified AR SAVI patients leads to AD SAVI c.842G>A p.(Arg281Gln). Patients with *de novo* or inherited c.842G>A p.(Arg281Gln) heterozygosity present with IFN activation and typical SAVI features such as ILD, failure to thrive, cough, dyspnea, and cutaneous necrotizing vasculitis (34–37). In some of these cases, poly-arthralgia, granulomatous hepatitis, respiratory distress, hypertension, nausea, vomiting, recurrent infections, and hypoxemia have been reported. However, presentation age of onset, and treatment responses after JAK inhibitor applications in patients carrying heterozygous c.842G>A p.(Arg281Gln) varied strongly (34–37). A 37-year-old man carrying a *de novo* heterozygous c.842G>A p.(Arg281Gln) variant displayed onset at adulthood of ILD, dyspnea, hypoxemia, severe cough, and clubbing. He received Ruxolitinib with poor response: passed away four months after treatment due to progressive ILD and heart failure (37). Thus far, it is unclear why distinct *STING1* variants in the same position can lead to AD or AR SAVI.

Our data does not suggest a clear difference in AR SAVI with regard to disease severity at onset as compared to AD SAVI. The mean age of AR SAVI onset of all cases we analyzed was 3.6 months (Stdev 2.6; range 0.5-8), and all children displayed first symptoms during infancy, whereas in AD SAVI phenotypes vary from severe skin and lung disease with onset during the first weeks of life (2) to adulthood onset, comparably mild lupus-like syndrome (38). The clinical findings in our newly identified patients included ILD with tachypnea and intermittent hypoxemia, and failure to thrive, which are in line with previously reported AD and AR cases of SAVI (13, 14, 34–37). Also, our findings on auto-antibody positivity as well as elevated inflammation and IFN-activation markers are in concordance with previous SAVI case reports (2, 38, 39). While vasculitis skin rash is typical for AD and AR SAVI (2, 13, 14, 38, 39), one of our patients displayed no skin vasculitis until the age of six years, which is atypical but has been previously reported in AD SAVI (35, 37, 40). Our patient 3 suffered from significant bullous skin disease on the face and extremities in infancy, suggesting a vasculitis lesion, but later recovered completely with residual scars, while the sister, patient 4, never showed any abnormalities on the skin. Just as it may be that skin lesions develop later, there are some SAVI patients in whom there is exclusively a pulmonary phenotype.

Our and other data on SAVI patients suggest that in addition to genetic factors disease manifestation and severity depend on other factors. Several authors have also claimed that patients with the inherited c.463G>A p.(Val155Met) variant in *STING1* have a less severe disease course than patients with the *de novo* c.463G>A p.(Val155Met) variant (2). Whether this is actually a result of the inheritance pattern, or caused by environmental factors, lifestyle and possibly ethical background, or whether additional polymorphisms are *in trans* has not been shown. There are several publications on the genetics of human *STING1* polymorphisms, which occur in more than 1% of the population, and their potential impact on human health has been discussed (41–44). Therefore, we screened our patients for the well-known and much-discussed *STING1* polymorphisms R71H, G230A, R232H, R293Q, V48V, HAQ (R71H-G230A-R293Q), and AQ (G230A-R293Q). HAQ, for example, is considered a null allele and therefore could affect disease progression in patients with AD or AR SAVI (45). Three of our four patients were homozygous for R232. Patient 2 remains a carrier of the V48V polymorphism. No other polymorphisms were detected in patients 1-3. Patient 4 was studied only by Sanger sequencing. Thus, in our patients an association of *STING1* polymorphisms with disease severity cannot be demonstrated and remains the subject of further studies.

Here we detail treatment regimens and response in all thus far described AR cases of SAVI. We show that treatment with hydroxychloroquine and high-dose methylprednisolone courses in first case may have beneficial effects on mitigation of lung disease prior to the diagnosis of SAVI. In the second patient, methylprednisolone courses had positive effect on skin lesions while not affecting ILD-related features of the disease. In the third patient, methylprednisolone pulse was able to improve the adynamic state and release arthralgia. In the fourth patient, treatment was not started yet. Type I IFNs induced by STING activation bind to receptors IFNAR1/2, subsequently activating the Janus kinases (JAK)/signal transducer and activator of transcription (STAT) pathway. In this context, JAK inhibitors have been applied in SAVI patients (9). The selective oral JAK1/2 inhibitor ruxolitinib showed marked positive effects on lung and cutaneous pathologies in AD SAVI without significant side effects (46). However, tofacitinib, an oral JAK1/3 inhibitor, have failed to inhibit dsDNA-activated, STING-dependent IRF3 phosphorylation in an *in vitro* SAVI model due to pathogenic variants located outside STING´s dimerization domain (40). Also, ruxolitinib have led to marked improvement of the pulmonary phenotype in a preschooler with AD SAVI caused by a pathogenic variant outside of the dimerization domain, but an evolving nasal septal erosion indicated incomplete control of disease (47). Wang et al. have reported three patients from one family with AD SAVI caused by c.842G>A p.(Arg281Gln), a variant outside of the dimerization domain (37). These patients have suffered from severe SAVI-related ILD, and while two of them have received ruxolitinib with poor response: one patient has passed away four months after treatment due to progressive ILD and heart failure. Population pharmacokinetics and pharmacodynamic analyses of baricitinib in patients with SAVI, and chronic atypical neutrophilic dermatosis with lipodystrophy and elevated temperature (CANDLE) showed a dose-dependent decrease in IFN biomarkers, which indicated the effect of baricitinib on type I IFN signaling (48). But the reduction of the IFN score was more significant in CANDLE patients, which has possibly been related to STING-independent intracellular signaling (49) rather than STING-dependent signaling in SAVI patients (2). Moreover, the half-life of baricitinib in patients weighing less than 40 kg was shorter than that in adult subjects, which raised the necessity of more frequent dosing (up to four times daily especially for patients weighing <20 kg) (48). Considering the severe disease manifestations in SAVI, a higher dose of baricitinib and weight-based dosing regimen has been recommended in pediatric patients (48, 50). With regard to the application of JAK-inhibitor therapy, it is too soon to evaluate treatment effects comprehensively in our newly identified cases of AR SAVI. We observed no negative treatment effects in our index patients. Of note, none of the applied treatment strategies in our cases had an effect on the patients´ IFN signature. Thus far, little systematic data on treatment regimens and clinical responses in SAVI is available (6, 7). Other therapeutics, such as TBK1 antagonists or AKT/AMPK inhibitors may be able to stop SAVI progression (40, 51), and it has been reported that GSK 690693 (AKT/AMPK inhibitor) but not tofacitinib (JAK inhibitor) were able to inhibit dsDNA activated, STING-dependent IRF3 phosphorylation in human fibroblast cells (hTERT-BJ1) expressing p.(Arg284Ser) *in vitro* (40). Nevertheless, further studies are required for those therapies. In this context, we would like to advocate for implementing all clinical and treatment data available in this rare, severe disease into international databases such as the one provided by the European children´s ILD network (child.eu where our cases were implemented) to pave the way for systematic clinical trials in the future.

Our work has certain limitations. For example, due to the refugee history of our first case, we were unable to comprehensively analyze medical records from the first years of this boy´s life, which may have led to an underreporting of early SAVI symptoms in this patient. The same limitation applies to the clinical data gathered from our literature research. In some of these cases (13, 14), for example features of ILD or other SAVI manifestations may have been underreported. Another limitation of our work is that we were unable to comprehensively perform lung phenotyping in all heterozygous carriers of c.841C>T p.(Arg281Trp). We are certain that all heterozygous carriers identified by our genotyping analyses were free of pulmonary complaints and did not display increased IFN activation. Also, no pulmonary disease was reported in the wider family of both cases. We did, however, not perform CCT scanning and in-depth immunological analyses in all heterozygous family members and can thus not exclude a mild effect of c.841C>T p.(Arg281Trp) which may occur in later stages of life. However, we find this highly improbable and strongly advocate for complete penetrance of c.841C>T p.(Arg281Trp) related SAVI.

In summary, we here present four novel cases of c.841C>T p.(Arg281Trp) related SAVI and show that SAVI may present as an AR trait. Here we deliver comprehensive data on all thus far identified AR SAVI cases and treatment regimens and responses. In public databases such as OMIM, SAVI remains to be listed as an exclusive AD trait. We hope our novel cases and comprehensive description of all thus far described carriers of biallelic c.841C>T p.(Arg281Trp) contribute to change this view and facilitate early diagnosis in this rare, severe disease.

## Data availability statement

The datasets for this article are not publicly available due to concerns regarding participant/patient anonymity. Requests to access the datasets should be directed to the corresponding author.

## Ethics statement

The Ethics Committee of LMU Munich, Germany, reviewed and approved the register (Project-No.: 111-13, dated 10 December 2013) and publication of results thereof (20-329, 30 October 2020). Written informed consent to participate in this study was provided by the participants’ legal guardian/next of kin. Written informed consent was obtained from the minor(s)’ legal guardian/next of kin for the publication of any potentially identifiable images or data included in this article.

## Author contributions

RW and SH analyzed genetic data; RW, SH, GS, LO, and CHe supported data interpretation; JF, IZ, DJ, ML-K, CW, NL, FS, CK, NS, MG, and MW provided samples and clinical data; LO, CHe, DH, MS, FS, and MW collected information on family histories; RW, SH, MW, CHa, CD, JF, FS, and DS wrote the manuscript; DS, BS, BA, MW, and SH designed and supervised the study. All authors commented to the manuscript and approved the submitted version.

## Funding

This study is funded by the Deutsche Forschungsgemeinschaft (DFG, German Research Foundation) under Germany’s Excellence Strategy – EXC 2155 – project number 390874280. Rensheng Wan holds a DAAD scholarship as part of the GSSP (Graduate School Scholarship Programme).

## Acknowledgments

The authors would like to thank the cooperation from the patients and their families. The authors sincerely thank Bernd Haermeyer and Michaela Losch for their excellent technical assistance.

## Conflict of interest

The authors declare that the research was conducted in the absence of any commercial or financial relationships that could be construed as a potential conflict of interest.

## Publisher’s note

All claims expressed in this article are solely those of the authors and do not necessarily represent those of their affiliated organizations, or those of the publisher, the editors and the reviewers. Any product that may be evaluated in this article, or claim that may be made by its manufacturer, is not guaranteed or endorsed by the publisher.

## References

[1] Barber GN. Sting: Infection, inflammation and cancer. Nat Rev Immunol (2015) 15(12):760–70. doi: 10.1038/nri3921 PMC5004891 26603901

[2] Liu Y Jesus AA Marrero B Yang D Ramsey SE Sanchez GAM. Activated sting in a vascular and pulmonary syndrome. N Engl J Med (2014) 371(6):507–18. doi: 10.1056/NEJMoa1312625 PMC4174543 25029335

[3] Ablasser A Chen ZJ. Cgas in action: Expanding roles in immunity and inflammation. Science (2019) 363(6431):eaat8657. doi: 10.1126/science.aat8657 30846571

[4] Decout A Katz JD Venkatraman S Ablasser A. The cgas–sting pathway as a therapeutic target in inflammatory diseases. Nat Rev Immunol (2021) 21(9):548–69. doi: 10.1038/s41577-021-00524-z PMC8029610 33833439

[5] Balci S Ekinci RMK de Jesus AA Goldbach-Mansky R Yilmaz M. Baricitinib experience on sting-associated vasculopathy with onset in infancy: A representative case from Turkey. Clin Immunol (2020) 212:108273. doi: 10.1016/j.clim.2019.108273 31626957

[6] Li W Wang W Wang W Zhong L Gou L Wang C. Janus kinase inhibitors in the treatment of type I interferonopathies: A case series from a single center in China. Front Immunol (2022) 13:825367. doi: 10.3389/fimmu.2022.825367 35418997 PMC8995420

[7] Gómez-Arias PJ Gómez-García F Hernández-Parada J Montilla-López AM Ruano J Parra-Peralbo E. Efficacy and safety of janus kinase inhibitors in type I interferon-mediated monogenic autoinflammatory disorders: A scoping review. Dermatol Ther (Heidelb) (2021) 11(3):733–50. doi: 10.1007/s13555-021-00517-9 PMC8163936 33856640

[8] Infevers: An online database for autoinflammatory mutations (Accessed March 15, 2022).

[9] David C Frémond ML. Lung inflammation in sting-associated vasculopathy with onset in infancy (Savi). Cells (2022) 11(3):318. doi: 10.3390/cells11030318 35159128 PMC8834229

[10] Landrum MJ Lee JM Benson M Brown GR Chao C Chitipiralla S. Clinvar: Improving access to variant interpretations and supporting evidence. Nucleic Acids Res (2018) 46(D1):D1062–d7. doi: 10.1093/nar/gkx1153 PMC5753237 29165669

[11] Guffroy A Dieudonné Y Gies V Danion F. Complex allele with additive gain-of-Function Sting1 variants in a patient with cavitating lung lesions and aspergillosis. J Clin Immunol (2022). doi: 10.1007/s10875-022-01284-8 35556195

[12] Seo J Kang JA Suh DI Park EB Lee CR Choi SA. Tofacitinib relieves symptoms of stimulator of interferon genes (Sting)-associated vasculopathy with onset in infancy caused by 2 *De novo* variants in Tmem173. J Allergy Clin Immunol (2017) 139(4):1396–9.e12. doi: 10.1016/j.jaci.2016.10.030 28041677

[13] Lin B Berard R Al Rasheed A Aladba B Kranzusch PJ Henderlight M. A novel Sting1 variant causes a recessive form of sting-associated vasculopathy with onset in infancy (Savi). J Allergy Clin Immunol (2020) 146(5):1204–8.e6. doi: 10.1016/j.jaci.2020.06.032 32673614 PMC8461559

[14] Alghamdi MA Mulla J Saheb Sharif-Askari N Guzmán-Vega FJ Arold ST Abd-Alwahed M. A novel biallelic Sting1 gene variant causing savi in two siblings. Front Immunol (2020) 11:599564. doi: 10.3389/fimmu.2020.599564 33488593 PMC7820697

[15] Amberger JS Bocchini CA Schiettecatte F Scott AF Hamosh A. Omim.Org: Online mendelian inheritance in man (Omim®), an online catalog of human genes and genetic disorders. Nucleic Acids Res (2015) 43(Database issue):D789–98. doi: 10.1093/nar/gku1205 PMC4383985 25428349

[16] Megsap - a medical genetics sequence analysis pipeline (Accessed June 07, 2021).

[17] Karczewski KJ Francioli LC Tiao G Cummings BB Alföldi J Wang Q. The mutational constraint spectrum quantified from variation in 141,456 humans. Nature (2020) 581(7809):434–43. doi: 10.1038/s41586-020-2308-7 PMC7334197 32461654

[18] Robinson JT Thorvaldsdóttir H Wenger AM Zehir A Mesirov JP. Variant review with the integrative genomics viewer. Cancer Res (2017) 77(21):e31–e4. doi: 10.1158/0008-5472.Can-17-0337 PMC5678989 29092934

[19] Cooper GM Stone EA Asimenos G Green ED Batzoglou S Sidow A. Distribution and intensity of constraint in mammalian genomic sequence. Genome Res (2005) 15(7):901–13. doi: 10.1101/gr.3577405 PMC1172034 15965027

[20] Kumar P Henikoff S Ng PC. Predicting the effects of coding non-synonymous variants on protein function using the sift algorithm. Nat Protoc (2009) 4(7):1073–81. doi: 10.1038/nprot.2009.86 19561590

[21] Adzhubei I Jordan DM Sunyaev SR. Predicting functional effect of human missense mutations using polyphen-2. Curr Protoc Hum Genet (2013) Chapter 7: Unit 7.20. doi: 10.1002/0471142905.hg0720s76 PMC4480630 23315928

[22] Shihab HA Gough J Cooper DN Day IN Gaunt TR. Predicting the functional consequences of cancer-associated amino acid substitutions. Bioinformatics (2013) 29(12):1504–10. doi: 10.1093/bioinformatics/btt182 PMC3673218 23620363

[23] Rentzsch P Witten D Cooper GM Shendure J Kircher M. Cadd: Predicting the deleteriousness of variants throughout the human genome. Nucleic Acids Res (2019) 47(D1):D886–d94. doi: 10.1093/nar/gky1016 PMC6323892 30371827

[24] Ioannidis NM Rothstein JH Pejaver V Middha S McDonnell SK Baheti S. Revel: An ensemble method for predicting the pathogenicity of rare missense variants. Am J Hum Genet (2016) 99(4):877–85. doi: 10.1016/j.ajhg.2016.08.016 PMC5065685 27666373

[25] Richards S Aziz N Bale S Bick D Das S Gastier-Foster J. Standards and guidelines for the interpretation of sequence variants: A joint consensus recommendation of the American college of medical genetics and genomics and the association for molecular pathology. Genet Med (2015) 17(5):405–24. doi: 10.1038/gim.2015.30 PMC4544753 25741868

[26] de Jesus AA Hou Y Brooks S Malle L Biancotto A Huang Y. Distinct interferon signatures and cytokine patterns define additional systemic autoinflammatory diseases. J Clin Invest (2020) 130(4):1669–82. doi: 10.1172/jci129301 PMC7108905 31874111

[27] Wolf C Brück N Koss S Griep C Kirschfink M Palm-Beden K. Janus kinase inhibition in complement component 1 deficiency. J Allergy Clin Immunol (2020) 146(6):1439–42.e5. doi: 10.1016/j.jaci.2020.04.002 32325142

[28] Gruber C Martin-Fernandez M Ailal F Qiu X Taft J Altman J. Homozygous Stat2 gain-of-Function mutation by loss of Usp18 activity in a patient with type I interferonopathy. J Exp Med (2020) 217(5):e20192319. doi: 10.1084/jem.20192319 32092142 PMC7201920

[29] Drutman SB Haerynck F Zhong FL Hum D Hernandez NJ Belkaya S. Homozygous Nlrp1 gain-of-Function mutation in siblings with a syndromic form of recurrent respiratory papillomatosis. Proc Natl Acad Sci U.S.A. (2019) 116(38):19055–63. doi: 10.1073/pnas.1906184116 PMC6754618 31484767

[30] Pandita S Khullar D Saxena R Verma IC. Autosomal dominant polycystic kidney disease: Presence of hypomorphic alleles in Pkd1 gene. Indian J Nephrol (2018) 28(6):482–4. doi: 10.4103/ijn.IJN_236_17 PMC6309388 30647506

[31] Vujic M Heyer CM Ars E Hopp K Markoff A Orndal C. Incompletely penetrant Pkd1 alleles mimic the renal manifestations of arpkd. J Am Soc Nephrol (2010) 21(7):1097–102. doi: 10.1681/asn.2009101070 PMC3152226 20558538

[32] Rossetti S Kubly VJ Consugar MB Hopp K Roy S Horsley SW. Incompletely penetrant Pkd1 alleles suggest a role for gene dosage in cyst initiation in polycystic kidney disease. Kidney Int (2009) 75(8):848–55. doi: 10.1038/ki.2008.686 PMC2813773 19165178

[33] Pommié C Levadoux S Sabatier R Lefranc G Lefranc MP. Imgt standardized criteria for statistical analysis of immunoglobulin V-region amino acid properties. J Mol Recognit (2004) 17(1):17–32. doi: 10.1002/jmr.647 14872534

[34] Melki I Rose Y Uggenti C Van Eyck L Frémond ML Kitabayashi N. Disease-associated mutations identify a novel region in human sting necessary for the control of type I interferon signaling. J Allergy Clin Immunol (2017) 140(2):543–52.e5. doi: 10.1016/j.jaci.2016.10.031 28087229

[35] Li J An S Du Z. Familial interstitial lung disease caused by mutation of the Sting1 gene. Front Pediatr (2020) 8:543. doi: 10.3389/fped.2020.00543 33014937 PMC7505928

[36] Volpi S Insalaco A Caorsi R Santori E Messia V Sacco O. Efficacy and adverse events during janus kinase inhibitor treatment of savi syndrome. J Clin Immunol (2019) 39(5):476–85. doi: 10.1007/s10875-019-00645-0 PMC7086512 31144250

[37] Wang Y Wang F Zhang X. Sting-associated vasculopathy with onset in infancy: A familial case series report and literature review. Ann Transl Med (2021) 9(2):176. doi: 10.21037/atm-20-6198 33569478 PMC7867893

[38] Jeremiah N Neven B Gentili M Callebaut I Maschalidi S Stolzenberg MC. Inherited sting-activating mutation underlies a familial inflammatory syndrome with lupus-like manifestations. J Clin Invest (2014) 124(12):5516–20. doi: 10.1172/jci79100 PMC4348945 25401470

[39] Clarke SL Pellowe EJ de Jesus AA Goldbach-Mansky R Hilliard TN Ramanan AV. Interstitial lung disease caused by sting-associated vasculopathy with onset in infancy. Am J Respir Crit Care Med (2016) 194(5):639–42. doi: 10.1164/rccm.201510-2102LE PMC5027210 27585386

[40] Konno H Chinn IK Hong D Orange JS Lupski JR Mendoza A. Pro-inflammation associated with a gain-of-Function mutation (R284s) in the innate immune sensor sting. Cell Rep (2018) 23(4):1112–23. doi: 10.1016/j.celrep.2018.03.115 PMC6092751 29694889

[41] Patel S Jin L. Tmem173 variants and potential importance to human biology and disease. Genes Immun (2019) 20(1):82–9. doi: 10.1038/s41435-018-0029-9 PMC6212339 29728611

[42] Yi G Brendel VP Shu C Li P Palanathan S Cheng Kao C. Single nucleotide polymorphisms of human sting can affect innate immune response to cyclic dinucleotides. PloS One (2013) 8(10):e77846. doi: 10.1371/journal.pone.0077846 24204993 PMC3804601

[43] Nissen SK Pedersen JG Helleberg M Kjær K Thavachelvam K Obel N. Multiple homozygous variants in the sting-encoding Tmem173 gene in hiv long-term nonprogressors. J Immunol (2018) 200(10):3372–82. doi: 10.4049/jimmunol.1701284 29632140

[44] Lubbers JM Koopman B de Klerk-Sluis JM van Rooij N Plat A Pijper H. Association of homozygous variants of Sting1 with outcome in human cervical cancer. Cancer Sci (2021) 112(1):61–71. doi: 10.1111/cas.14680 33040406 PMC7780010

[45] Patel S Blaauboer SM Tucker HR Mansouri S Ruiz-Moreno JS Hamann L. The common R71h-G230a-R293q human Tmem173 is a null allele. J Immunol (2017) 198(2):776–87. doi: 10.4049/jimmunol.1601585 PMC5225030 27927967

[46] Frémond ML Rodero MP Jeremiah N Belot A Jeziorski E Duffy D. Efficacy of the janus kinase 1/2 inhibitor ruxolitinib in the treatment of vasculopathy associated with Tmem173-activating mutations in 3 children. J Allergy Clin Immunol (2016) 138(6):1752–5. doi: 10.1016/j.jaci.2016.07.015 27554814

[47] Saldanha RG Balka KR Davidson S Wainstein BK Wong M Macintosh R. A mutation outside the dimerization domain causing atypical sting-associated vasculopathy with onset in infancy. Front Immunol (2018) 9:1535. doi: 10.3389/fimmu.2018.01535 30038614 PMC6047589

[48] Kim H Brooks KM Tang CC Wakim P Blake M Brooks SR. Pharmacokinetics, pharmacodynamics, and proposed dosing of the oral Jak1 and Jak2 inhibitor baricitinib in pediatric and young adult candle and savi patients. Clin Pharmacol Ther (2018) 104(2):364–73. doi: 10.1002/cpt.936 PMC6089664 29134648

[49] Brehm A Liu Y Sheikh A Marrero B Omoyinmi E Zhou Q. Additive loss-of-Function proteasome subunit mutations in Candle/Praas patients promote type I ifn production. J Clin Invest (2015) 125(11):4196–211. doi: 10.1172/jci81260 PMC4639987 26524591

[50] Cetin Gedik K Lamot L Romano M Demirkaya E Piskin D Torreggiani S. The 2021 European alliance of associations for Rheumatology/American college of rheumatology points to consider for diagnosis and management of autoinflammatory type I interferonopathies: Candle/Praas, savi, and ags. Arthritis Rheumatol (2022) 74(5):735–51. doi: 10.1002/art.42087 35315249

[51] Hasan M Yan N. Therapeutic potential of targeting Tbk1 in autoimmune diseases and interferonopathies. Pharmacol Res (2016) 111:336–42. doi: 10.1016/j.phrs.2016.04.008 PMC5703047 27353409

