# Supplementary material for: Phenotypic spectrum in recessive STING-associated vasculopathy with onset in infancy: Four novel cases and analysis of previously reported cases
Source: Front Immunol. 2022 Oct 6;13:1029423. doi: 10.3389/fimmu.2022.1029423 (PMC9583393; doi:10.3389/fimmu.2022.1029423)
Supplement: Supplementary file 1 [file DataSheet_1.pdf]

frontiers

Supplementary Material

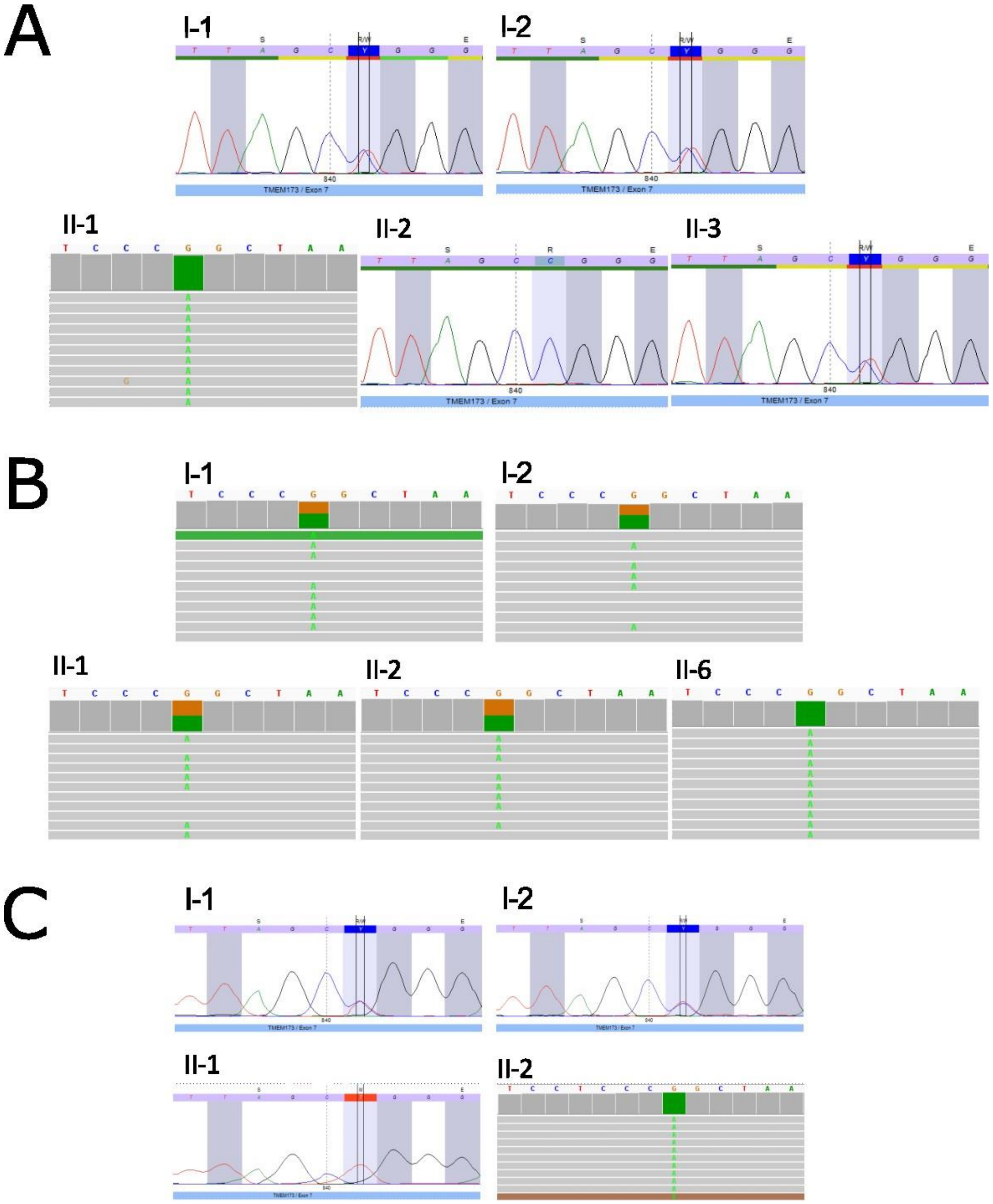

Figure S1 | Segregation analysis for three families.

(A) Segregation analysis for family 1 by whole exome sequencing and Sanger sequencing. (**B**) Segregation analysis for family 2 by whole exome sequencing and whole genome sequencing. (**C**) Segregation analysis for family 3 by whole genome sequencing and Sanger sequence.

Table S1. Individual 7 IFN-related gene expression in three families (relative mRNA expression).

	**Members**	***IFI27***	***IFI44***	***IFI44L***	***IFIT1***	***ISG15***	***RSAD2***	***SIGLEC1***	**IFN-Score**
Family 1	I-1 (Father)	1,074	-1,413	-0,913	-0,150	-1,473	0,135	-1,008	**-3,747**
	I-2 (Mother)	4,597	-1,300	-0,081	6,620	-1,459	11,661	-0,873	**19,164**
	II-1 (Patient 1) (6 months after JAK-I treatment)	89,424	0,429	15,078	15,165	-1,270	19,386	-0,387	**137,825**
	II-1 (Patient 1) (7 months after JAK-I treatment)	659,869	0,113	16,575	38,290	1,175	92,438	1,413	**809,871**
Family 2	I-1 (Father)	-0,601	-1,425	-0,963	-0,133	-1,361	-0,939	-0,647	**-6,067**
	II-2 (Brother)	1,486	-1,336	0,370	6,779	-1,150	6,138	-0,322	**11,965**
	II-6 (Patient 2)	321,236	0,964	20,271	25,464	-0,282	76,358	0,454	**444,466**
Family 3	II-1 (Patient 4)	4,197	23,617	137,556	644,621	98,365	1220,366	6,865	**2135,588**
	II-2 (Patient 3)	11,696	57,670	267,005	559,203	44,426	2083,289	0,782	**3024,071**
